# Stability and Exsolution of Sr_0.98_Ti_0.7_Fe_0.25_Ni_0.05_O_3_ for the Oxygen Evolution Reaction in an Alkaline Environment

**DOI:** 10.1021/jacs.5c05748

**Published:** 2025-07-16

**Authors:** Fabian Luca Buchauer, SangWoo Kim, So̷ren Bredmose Simonsen, Roxy Lee, WooChul Jung, Christodoulos Chatzichristodoulou

**Affiliations:** † Department of Energy Conversion and Storage, 5205 Technical University of Denmark (DTU), Building 310, Fysikvej, Lyngby 2800, Denmark; ‡ 34968 Korea Advanced Institute of Science and Technology (KAIST), 291 Daehak-ro, Yuseong-gu, Daejeon 34141, Republic of Korea; ¶ 26725 Seoul National University (SNU), 1, Gwanak-ro, Gwank-gu, Seoul 08826, Republic of Korea

## Abstract

Developing stable, critical raw material lean catalysts for the OER is essential for low-cost green hydrogen production. In this study, dense, polished pellets of Sr_0.98_Ti_0.7_Fe_0.25_Ni_0.05_O_3_ (STFNO) were employed as a model system to investigate intrinsic OER activity and stability under industrially relevant conditions. The well-defined surface and controlled interface between the sample and electrolyte represent a more fundamental approach to studying catalytic performance. Testing was conducted at elevated temperatures up to 150 °C and at a pressure of 50 bar, offering unique insights into catalytic activity and stability under harsh conditions. STFNO demonstrated excellent stability during prolonged testing and high catalytic activity. This study also explored the role of exsolution in enhancing OER performance under industrially relevant conditions. Homogeneous (Ni,Fe)­O_*x*_ nanoparticles, exsolved at 600 °C, yielded a notably low overpotential of 199 mV at 75 °C. These findings provide valuable insights into the activity and stability of STFNO as an industrially viable catalyst.

## Graphical abstract

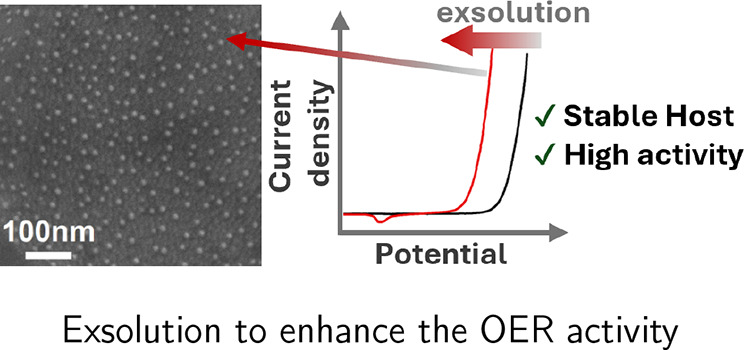

## Introduction

Electrolysis is a key technology for reducing green house gas emissions as it is a large-scale solution for storing renewable electricity and providing green hydrogen. Electrolysis cells are composed of two half-cell reactions: the oxygen evolution reaction (OER) and the hydrogen evolution reaction (HER). Among the different electrolysis technologies, alkaline electrolysis is the most established technology at a large scale. However, the efficiency is severely constrained by the sluggish oxygen evolution reaction (OER), which has been the subject of extensive research. Industrial alkaline electrolyzers are typically operated at temperatures of 60–90 °C, using relatively high electrolyte concentrations of 6–10 M, and are preferably pressurized.^1,2^ Increasing the pressure is beneficial, as most applications, such as mobility infrastructure, ammonia synthesis, methanol synthesis, grid injection, and the transportation of hydrogen, require pressurized hydrogen. The anticipated lower cost of electrochemical over mechanical compression makes pressurized cell operation particularly advantageous. Increasing the operating temperature is extremely beneficial in terms of thermodynamics, ionic conductivity of the electrolyte, and electrode kinetics.^3–8^ Nevertheless, the stability of OER catalysts poses a significant challenge at elevated temperatures, already of concern at low temperatures. However, catalyst studies typically do not consider elevated temperature or pressurized cell operation.

Commonly studied OER catalyst materials belong to three distinct groups: layered double hydroxides,^9–11^ spinel-type oxides,^12^ and perovskite oxides.^13,14^ Members of these groups have demonstrated exceptionally high activities during lab-scale testing in dilute electrolyte (0.1 or 1 M), atmospheric conditions (ambient temperature and pressure), and low current densities. These conditions hardly reflect the harsh operating conditions inside a real electrolyzer, as has been pointed out by Siegmund et al. and Ehlers et al.^8,15^ Stability studies of catalyst materials under more industrially relevant test conditions have shown instability for members of all the above-mentioned material classes that appeared promising under lab-scale testing.^16–21^ Furthermore, a true surface normalization of the activities is seldom attempted, which makes it challenging to compare reported activities from different studies.

In particular, perovskite materials (ABO3, where A and B are cations) have gained significant attention due to their ability to host a wide variety of elements at both the A- and B-sites, enabling the engineering of their properties. Studies of highly active perovskites have shown overpotentials of 260–370 mV at 10 mA cm^–2^ (geometric area normalized) in 0.1 M KOH at 25 °C.^13,14,22–28^ Several of the most active cobaltites have been reported to decompose, even at 25 °C, highlighting their inherent instability. Recent studies also reveal the decomposition of Ni-based perovskites and other La-based perovskites, like La_0.75_Sr_0.25_MnO_3−δ_ (LSMO), LaFeO_3−δ_ (LFO), (La_0.6_Sr_0.4_)_0.98_FeO_3−δ_ (LSFO), La_0.6_Sr_0.4_Fe_0.8_Co_0.2_O_3−δ_ (LSCFO), and (La_0.6_Sr_0.4_)_0.99_CoO_3−δ_ (LSCO), under industrially relevant conditions, underscoring the stability challenges of these materials.^17,21^

Exsolution can be used to increase the catalytic activity of perovskites. It refers to the spontaneous formation of metal catalyst nanoparticles on the surface of a host structure by partial decomposition of the host oxide during, e.g., a reducing heat treatment.^29–31^ This single-step process is primarily used in high-temperature catalysis to produce evenly dispersed and anchored nanoparticles on the surface of the host structure, preventing common catalyst degradation mechanisms.^32–34^ Exsolution results in a hybrid catalyst combining transition-metal nanoparticles and a metal oxide support. Studies of exsolution-based materials as OER catalysts in alkaline media have shown overpotentials in the range of 210–400 mV at 10 mA cm^–2^ (geometric area normalized) for the most active materials tested in 0.1 or 1 M KOH at 25 °C.^35–41^ However, in most cases, critical raw materials (CRM) are used on either the A- or B-site of the perovskite, which should preferably be avoided.

To overcome the presented challenges, Sr_1–*y*_Ti_0.95–*x*_Fe_*x*_Ni_0.05_O_3_-based catalysts were explored as a potentially stable and active CRM-lean catalyst alternative. Accelerated corrosion testing was used to determine the best Ti-to-Fe ratio for optimal stability. Dense, polished pellets of the catalyst material were used, allowing for low and comparable surface areas. The material’s activity and stability were evaluated at industrially relevant conditions (at temperatures from 25 to 150 °C, at 50 bar, and in 10 M KOH) using a pressurized autoclave test setup. To assess the electrochemical stability of STFNO at industrially relevant conditions, long-term testing at 10 mA cm^–2^ was performed for 200 h at 100 and 150 °C. Furthermore, a reducing heat treatment was used to enhance the catalytic activity of STFNO by exsolving (Ni,Fe)­O_*x*_ nanoparticles on the surface of the host oxide and tested up to 100 °C.

## Results and Discussion

### Accelerated Stability Testing

Buchauer et al. have shown that performing accelerated stability tests is an excellent way to identify suitable catalyst candidates that can withstand the harsh electrolysis conditions.^17^ Buchauer et al. and Adolphsen et al. have shown that the most researched lanthanide perovskites (La_0.75_Sr_0.25_MnO_3−δ_ (LSMO), LaFeO_3−δ_ (LFO), (La_0.6_Sr_0.4_)_0.98_FeO_3−δ_ (LSFO), (La_0.6_Sr_0.4_)_0.99_CoO_3−δ_ (LSCO), and LaNiO_3−δ_ (LNO)) all form secondary phases during accelerated stress testing at 200 °C.^17,21^ La_0.6_Sr_0.4_Fe_0.8_Co_0.2_O_3−δ_ (LSCFO) was the only material that did not exhibit chemical instability after accelerated stability testing at 200 °C; however, strong signs of decomposition were observed after prolonged electrochemical testing at 75 °C and 50 bar for 200 h in 10 M KOH.

Here, we tested the stability of five different STFNO compositions, Sr_0.95_Ti_0.95–*x*_Fe_*x*_Ni_0.05_O_3_ with *x* = 0.05; 0.25; 0.45; 0.65, and Sr_0.98_Ti_0.7_Fe_0.25_Ni_0.05_O_3_ by assessing whether phase decomposition of the pristine powders takes place after immersion for 72 h in 10 M KOH at 200 °C. A-site deficient compositions were chosen as it has been shown by Kwon et al. and Papargyriou et al. that A-site deficiency promotes B-site metal exsolution.^42,43^ The 5% A-site deficient samples all exhibited a small fraction of the NiO secondary phase in the pristine state, as can be seen in Figure S1. This is consistent with literature reports from Zhu et al., which have mentioned the difficulty in synthesizing STFNO with an A-site deficiency ≥5% without forming the NiO secondary phase.^44^ The 2% A-site deficient sample did not show any secondary phase in its pristine state.

The XRD results of the perovskite powders immersed at 200 °C for 72 h in 10 M KOH in an autoclave can be seen in Figure 1 and show a clear trend: with increasing Fe amount, the stability of the material decreases. STFNO containing lower Fe amounts (*x* = 0.05 and 0.25) showed no signs of instability, whereas STFNO with higher amounts of Fe (*x* = 0.45 and 0.65) showed clear signs of secondary phases of Fe_3_O_4_ and Sr_2_FeO_4_ appearing. After stability testing, the 2% A-site deficient sample showed the formation of a small amount of Fe_3_O_4_. Despite that, the 2% A-site deficient sample was chosen for further testing, as it showed both good stability and a pure phase in the pristine state.

**Figure 1:**
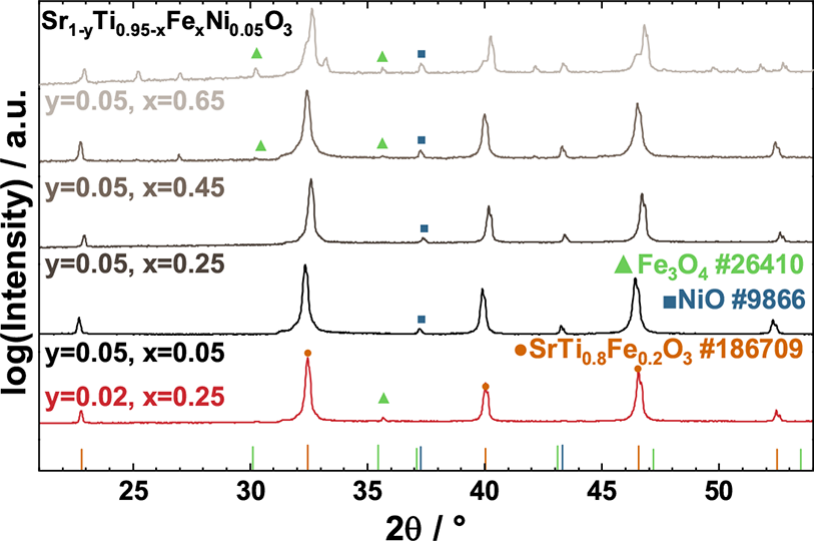
Stability screening of Sr_1–*y*_Ti_0.95–*x*_Fe_*x*_Ni_0.05_O_3_ perovskites with different Ti-to-Fe ratios and two different A-site deficiencies XRD patterns after immersion in 10 M KOH at 200 °C for 72 h in an autoclave.

### Activation of Pristine STFNO

The intrinsic OER activity of Sr_0.98_Ti_0.7_Fe_0.25_Ni_0.05_O_3_ (STFNO) at 50 bar and at various temperatures (25, 50, 75, 100, 125, and 150 °C) is shown in Figure 2A. It exhibits thermally activated kinetics, with a pronounced increase in performance from 25 to 50 °C. Furthermore, the overpotential at 10 mA cm^–2^ seems to decrease, when the pressure is increased from 1 to 50 bar by 11 to 68 mV, depending on the surface condition of the sample, as can be seen in Figure 2B1 and in Table S1.

**Figure 2:**
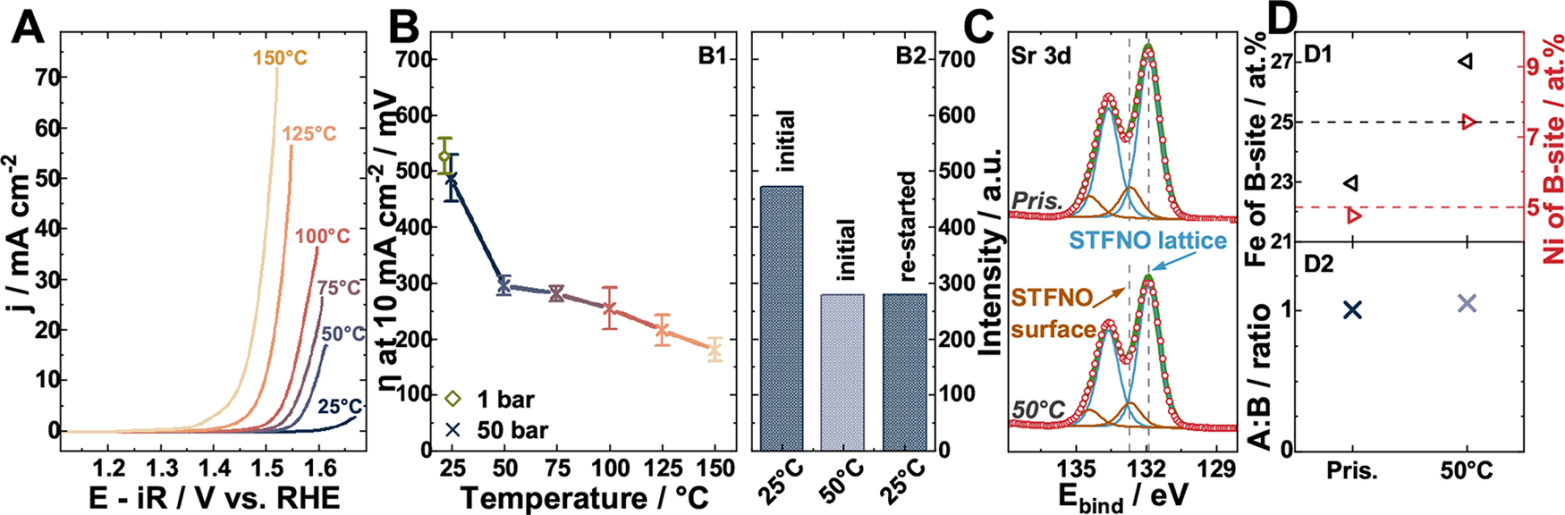
Electrochemical activity of STFNO at 25–150 °C in 10 M KOH. (A) Cathodic sweeps at a scan rate of 10 mV s^–1^ at 50 bar. (B) Overpotential development at the benchmark current density of 10 mA cm^–2^ (average of five measurements with the error bar representing the standard deviation of the mean) (B1) over whole temperature range B2) Overpotential of sample that was used for XPS surface activation study at 50 bar. (C) XPS Sr 3d narrow scan region. (D) XPS quantification (D1) Fe and Ni ratio of B-site (D2) A to B ratio.

The overpotential at 10 mA cm^–2^ shows a sharp decrease by 192 mV when the temperature is increased from 25 to 50 °C, a trend constantly seen for all five tested STFNO samples. To assess a possible surface chemical origin of the observed activation, one sample was tested only up to 50 °C, and XPS was carries out prior to and after testing. The XPS results of the Sr 3d region can be seen in Figure 2C and the compositional analysis in Figure 2D. The Sr 3d spectra reveal that there are Sr surface species both prior to and after testing at 50 °C. The surface component around 132.6 eV represents Sr-rich surface species that could originate from either Sr­(OH)_*x*_ or SrO, which are commonly identified on STO and STO-related surfaces.^45–50^ Figure 2D2 shows that after testing to 50 °C, the A/B ratio is unchanged. When restarting the test after XPS characterization, the sample showed a significantly lower overpotential; see Figure 2B2. The compositional analysis shown in Figure 2D1 revealed a slight increase in the Fe and Ni amounts on the B-site. The higher Ni and Fe amount could indicate the exposure of more BO-terminated surfaces, which are more active toward the OER.

The overpotential at 10 mA cm^–2^ follows a mild thermal activation thereon, with a value of 283 ± 14 mV at 75 °C and 50 bar, further decreasing to 183 ± 20 mV at 150 °C.

### Stability of Pristine STFNO

Long-term stability at industrially relevant operating conditions was tested at the benchmark current density of 10 mA cm^–2^ for 200 h at 50 bar at 100 and 150 °C. Where 100 °C is already on the higher end of the commercial operational window. Pressurization allows for operation beyond usual industrial conditions, as the electrolyte remains liquid; hence, long-term testing is also performed at 150 °C. Testing under these conditions (at higher operating temperatures and pressure) is of interest for advanced AEL and as an accelerated degradation probe.^8,15,51^ The test results can be seen in Figure 3A/B. Figure 3A displays the overpotential development over time.

Overall, the measurement at 100 °C exhibits an increasing overpotential from 300 to 320 mV in the first approximately 90 h of the test. After 91 h, a full characterization is performed, after which the overpotential stabilizes between 136 and 186 h at 315 mV. It should be noted that the overpotential undergoes a slow relaxation from approximately 250 mV toward 320 mV at the start of the measurement and after the short interruptions at 91 and 187 h. The first exponential part of the transient is attributed to the gradual establishment of a steady-state of oxygen saturation within the electrolyte after initiation of the OER and the second linear part to electrolyte polarization. The overpotential of 256 ± 37 mV was indeed determined during short-term testing. After 91 and 200 h of operation at 100 °C, 50 bar, 10 mA cm^–2^, a full characterization is performed, and the cathodic sweeps can be seen in Figure 3B. An increase in performance is observed as a shift of the cathodic sweeps by roughly 20 mV toward lower potentials. Both cathodic sweeps after 91 and 200 h exhibit a distinct reduction feature at 1.23 V vs RHE, which can be attributed to the Ni^3+^/Ni^2+^ redox transition.^11,52^ This redox feature was not present at the start of the prolonged electrolysis test.

The long-term stability test at 150 °C displays a reverse initial transient, with the overpotential decreasing from 300 to 180 mV within the first 6 h of operation. The observed decrease in overpotential during the first 6 h, despite stable temperature conditions, could potentially be attributed to the stabilization of the reference electrode, restructuring of the electrode surface, or stabilization of the electrode–electrolyte interface; however, the exact cause remains speculative. Then, the overpotential gradually increases from 190 ± 10 to 230 ± 15 mV. After the first 6 h, the overpotential is similar to the value of 180 ± 20 mV determined during short-term testing of 183 ± 20 mV. It is noted that the ca. 10 mV overpotential oscillations with hourly frequency observed for both tests are attributed to bubble formation and release. Similar to the long-term test at 100 °C, the slow increase in the overpotential is at least partly attributed to electrolyte polarization.

The catalyst was examined post-mortem using SEM, XRD, and XPS. Figure 3D exhibits the XRD results of the STFNO after 200 h at 100 and 150 °C. The results show that the overall bulk perovskite structure is retained in both cases. However, small amounts of NiO secondary phase were seen in the XRD pattern of the sample tested at 100 °C. The formation of Fe_3_O_4_ (or a mixed Ni–Fe oxide) cannot be excluded for the sample tested at 150 °C. The SEM analysis in Figure 3H showed an almost unchanged surface after testing at 100 °C (orange frame and Figure S4) compared to before (see Figure S3). The SEM analysis after testing at 150 °C (yellow frame and Figure S5) showed roughening of the surface compared to before (see Figure S3), seemingly due to particles having fallen off.

The ICP analysis in Figure 3G revealed that after testing at 100 °C for 200 h, the amount of Fe in the electrolyte is similar to the amount in the initial electrolyte of about 0.32 ppm, but small amounts of Sr (0.15 ppm) were found. After testing at 150 °C for 200 h, the amount of Fe is 10× times higher (3.2 ppm), and the amount of Sr has increased to 0.53 ppm. This indicates a significant amount of Fe and Sr leaching from the sample tested at 150 °C for 200 h. In Buchauer et al., cation dissolution was identified as a dominant degradation pathway, which ultimately can lead to the breakdown of the perovskite structure under OER conditions.^17^ However, it remains unclear whether Sr and Fe dissolution continues beyond 200 h or stabilizes once the electrolyte approaches saturation.

XPS was used to analyze possible surface changes after testing. The XPS narrow scan spectra can be seen in Figure 3C, and the quantification results can be seen in Figure 3E/F. The Sr 3d and O 1s spectra can be fitted by an STFNO lattice component at lower binding energies and surface-related components at higher binding energies. Deconvolution of the Fe 2p and Ni 2p peaks is not attempted due to the combined effects of spin–orbit splitting, multiplet splitting, shakeup satellites, and chemical state variations, making a reliable peak fit challenging. Furthermore, the Fe 2p peak coincides with the peak position of the Ni LMM auger peak, in addition to the Ni 2p signal overlapping with the Fe LMM auger, further complicating peak fitting.

**Figure 3:**
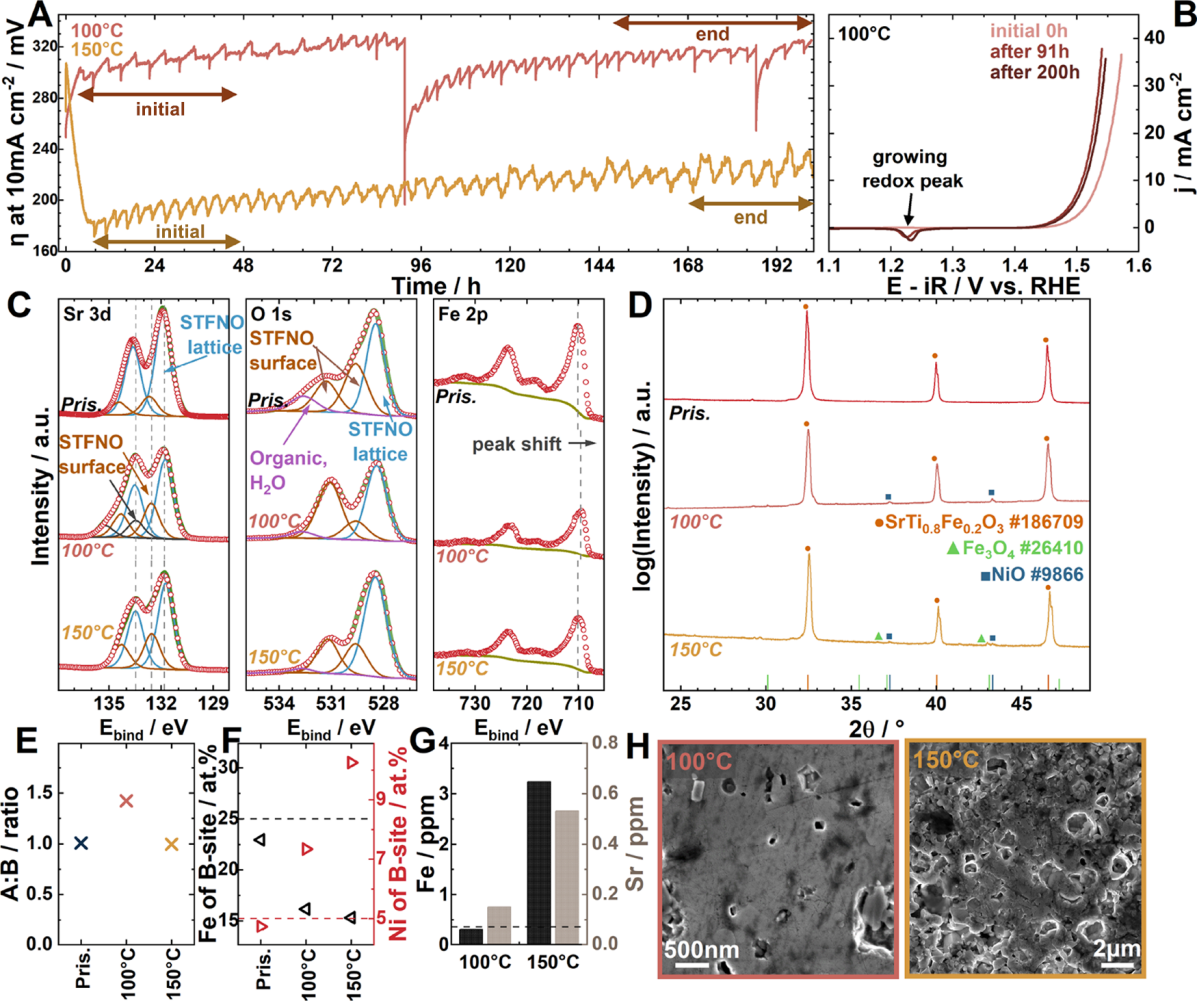
(A) Overpotential development at 10 mA cm^–2^ over the 200 h experiment at 100 and 150 °C. (B) Cathodic sweeps at start and after 91 and 200 h at 100 °C. (C) XPS narrow scan regions of STFNO before and after testing at 100 and 150 °C. (D) XRD results of the tested samples. (E) A to B ratio in the different samples from XPS. (F) Fe and Ni content of the B-site from XPS. (G) ICP of electrolyte after test (black dashed line indicates Fe impurities at the start). (H) Exemplary SEM images after testing for 200 h.

There is a degree of asymmetry to the Sr 3d spectra (raw spectra in Figure S13), which is well fitted with a second doublet 0.8 eV higher than the lattice doublet in all samples, except for the sample tested at 100 °C, which requires an additional component at higher BE. Strontium oxides and other mixed oxides containing strontium are anticipated to chemisorb both water and carbon dioxide, producing surface hydroxides and carbonates, although it is difficult to differentiate between SrO, Sr­(OH)_*x*_, and SrCO_3_ from the Sr 3d and O 1s binding energies.^53^ However, it is possible to identify SrCO_3_ from a peak around 289 eV in the C 1s spectra, which is present in the case of the 100 and 150 °C tested samples. Although it does not fully account for the area of the Sr surface components, it is likely that there is a contribution from carbonate species. It is also likely that there are Sr­(OH)_*x*_ species due to testing in KOH, which also caused the appearance of K 2p peaks.

The increase in surface species present in the Sr 3d spectra seen for the sample tested at 100 °C compared to the pristine sample can also be seen in the O 1s spectra. To identify the higher BE surface component in the Sr 3d spectra, the Pourbaix diagram of Sr from Pourbaix et al. was considered.^54^ It is possible that during operation, the conditions fall within the range of SrO_2_ formation. Consequently, the surface component could be attributed to SrO_2_, which could be expected to produce a component at higher binding energies, though no XPS reference data for SrO_2_ are available. The quantification results in Figure 3E reveal a surface enrichment with Sr, as the A to B ratio shifted from 1 to 1.4. This is in agreement with the Pourbaix diagram of Sr, in which the stability of the SrO_2_ phase at the electrolysis conditions is predicted. The Fe 2p spectral maximum exhibits a shift to lower binding energies, indicating a slight increase in the ratio of Fe^2+^/Fe^3+^; an enlarged plot of the Fe 2p spectral maximum displaying the observed shift can be found in Figure S13B.^48,55,56^ This is only considered to be a small increase due to the satellite peak observed at 719 eV being characteristic of Fe^3+^.^48^ Also, Figure 3F displays a strong decrease of Fe in the B-site from 23 atom % down to 16 atom %, accompanied by an increase in Ni in the B-site from 5 to 7 atom %. The enrichment of Ni shows similar levels, as reported in Figure 2D1. The harsh operating conditions at 100 °C place significant stress on the material, leading to the formation of a potentially stable (i.e., SrO_2_) inactive Sr surface phase and the leaching of Fe, both of which could contribute to the gradual increase in overpotential observed.

Turning to the sample tested at 150 °C for 200 h and considering the Pourbaix diagram of Sr, the decreased overpotential at 150 °C results in operation in the Sr­(OH)_*x*_ region; therefore, dissolution of Sr is expected. This was confirmed by the increased level of Sr in the electrolyte measured with ICP. A similar A to B ratio compared with the pristine sample was found after the test. The position of the Fe 2p spectral maximum was similar to the pristine sample, indicating a similar ratio of Fe^2+^/Fe^3+^. Figure 3F displays the Fe and Ni atomic concentrations of the B-site. The Fe ratio decreased from 23 to 15 atom %, maintaining a similar level as after testing at 100 °C for 200 h. On the other hand, the Ni concentration is increased from 5 to 10 atom %, a further increase from the 7 atom % level after testing for 200 h at 100 °C.

Dynamic surface reconstruction has been reported for Ba- and La-based cobaltites and nickelates, where the formation of self-assembled oxy­(hydroxide) layers under OER conditions, linked to the lattice oxygen evolution reaction (LOER), leads to activation of the perovskite.^14,35,57,58^ This is due to the dissolution and redeposition of A- and B-site cations, resulting in an active oxy­(hydroxide) surface phase. While a similar formation of a Ni,Fe oxy­(hydroxide) in our system is plausible, we do not have direct evidence of the formation of such a layer from XPS, XRD, or SEM.

Overall, the stability of STFNO can be summarized:

- Post-mortem analysis of the sample tested at 150 °C for 200 h revealed microstructural changes visible in SEM in the form of particles fallen off and a general roughening of the surface. XPS analysis showed a depletion of Fe from the surface and enrichment of Ni in the surface layer. Dissolution of Fe was confirmed with ICP. An increased amount of Sr was found in the electrolyte, which indicates the breakdown of the surface perovskite structure visible in the SEM analysis and confirmed by low amounts of secondary phase in XRD. The changing microstructure is likely responsible for at least part of the gradual increase in overpotential measured.
- Testing at 100 °C for 200 h showed an intact microstructure in the SEM analysis. XPS analysis revealed changes in the near-surface layer, notably the enrichment of Sr and Ni species and the depletion of Fe. This was accompanied by a small fraction of NiO secondary phase detected after testing. The results indicate a nearly intact perovskite surface after testing, with minor surface compositional changes.

### State-of-the-Art Comparison

Comparing intrinsic activities is difficult when the measurement conditions are different and the electrochemically active surface area are uncertain. Therefore, a comparison is only attempted with Buchauer et al. as they measured intrinsic activities under the same conditions and for dense, polished pellets with well-defined surface areas.^17^ Buchauer et al. measured the intrinsic activity of LFO and LSCFO, where both showed low overpotentials of approximately 300 mV at 10 mA cm^–2^ at 75 °C, 50 bar, in 10 M KOH.^17^ However, both materials exhibited instability over 200 h of operation at these conditions, with LSCFO’s overpotential decreased and stabilized at 280 mV, attributed to the breakdown of the perovskite and the formation of CoOOH. LFO’s overpotential increased and stabilized at 350 mV, with significant surface roughening, Fe depletion, and La enrichment. It should be noted that both materials contained a significant amount of CRM. In contrast, STFNO demonstrated much higher stability during testing at 100 °C for 200 h, maintaining an intact microstructure and only minor surface composition changes, with no significant degradation, stabilizing at an overpotential of 320 mV. This suggests that while LFO and LSCFO are active catalysts, STFNO is both active and reasonably stable, while containing less CRM, making it a more suitable candidate for industrial applications.

### Exsolution as Performance Booster

A prominent strategy to boost the OER performance of perovskite materials in alkaline electrolysis is via B-site cation exsolution.^35,38,59–61^ STFNO is a well-suited host for exsolution, as previously shown by Zhu et al.^44,62^ This is due to the A-site deficiency acting as an exsolution booster and the Ni reduction reaction having a negative Gibbs free energy at mildly reducing conditions, which makes Ni easily reducible from the perovskite lattice.^30^ However, STFNO has only ever been used in the field of solid oxide fuel cells (SOFC) as an anode material. Recently, Wang et al. has shown a strong boost in OER performance of Sr­(Ti,Fe)­O_3_ in AEC upon exsolution.^63^ Neagu et al. demonstrated how the reduction conditions influence the nanostructure of the exsolved particles from La_0.43_Ca_0.37_Ti_0.94_Ni_0.06_O_3_, showing that cubic or spherical nanoparticles can be obtained based on different conditions.^34^ In this study, different exsolution temperatures were tested while keeping the other parameters constant: a duration of 5 h in a dry atmosphere containing 4% H2/96% N2. Specifically, the exsolution of STFNO was carried out at temperatures of 600, 700, and 800 °C.

### Exsolution Characterization

The XRD patterns of the exsolved STFNO samples are shown in Figure 4A. After exsolution, the main peak of STFNO is shifted toward lower 2θ angels. This is to be expected and has previously been associated with oxygen loss and the reduction in the oxidation state of Fe in the perovskite due to a decrease in A-site substoichiometry.^62^ The size and distribution of the exsolved nanoparticles can be seen for each temperature in Figure 4B–D and are listed in Table 1. According to the SEM images, the exsolved particles are homogeneously distributed at the host surface for all three temperatures. The size of the nanoparticles increases with temperature, and the particle density decreases. This is in agreement with previous literature reports.^64,65^

**Figure 4:**
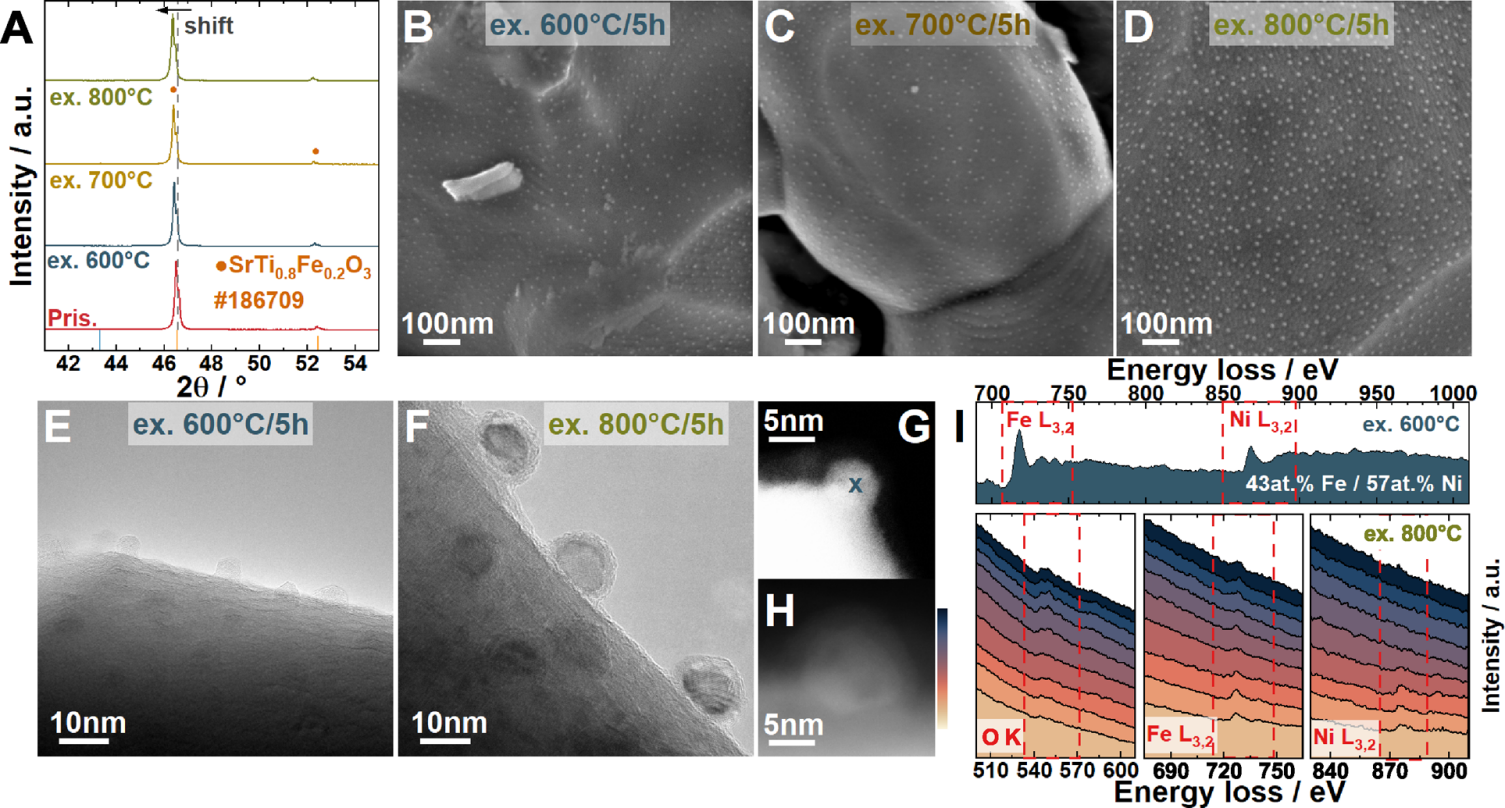
(A) XRD patterns of the STFNO sample after exsolution at 600–800 °C in diluted H2. (B–D) SEM images of STFNO exsolved at different temperature. (B) Ex.600 °C. (C) Ex.700 °C. (D) Ex.800 °C. (E) TEM image of exsolved particles at 600 °C. (F) TEM image of exsolved particles at 800 °C. (G) STEM image of particles (ex. at 600 °C) used for EELS analysis. (H) STEM image of particles (ex. at 800 °C) used for EELS linescan. (I) EELS spectra.

**Table 1:** Average Particle Size, Particle Density, and Their Corresponding Standard Deviation for Various Exsolution Temperatures

temperature (°C)	particle size (nm)	particle density (#/μm^2^)
600	8.1 ± 2.4	946 ± 30
700	9.2 ± 2.2	834 ± 44
800	10.5 ± 2.5	808 ± 14

The average particle size on the STFNO - Ex. 600 °C sample was 8.1 nm with a standard deviation of 2.4 nm. STFNO Excitation at 600 °C exhibited a high particle density of 946 #/μ m^2^. STFNO - Ex. 800 °C, on the other hand, had larger exsolved particles of 10.5 nm with a standard deviation of 2.5 nm and a reduced particle density of 808 #/μ m^2^. With increasing reduction temperature, the observed increase in particle size accompanied by a decrease in particle density suggests that exsolution occurs predominantly from the surface-near region, consistent with recent literature attributing this behavior to kinetic and thermodynamic constraints.^34,66^

Figure 4 shows TEM and HAADF-STEM images of the exsolved nanoparticles on the oxide support of STFNO - Ex.600 °C (Figure 4E/G) and STFNO - Ex.800 °C (Figure 4F/H). In both cases, the particles appear to be spherical and partly embedded into the perovskite surface, similar to what has been reported in Neagu et al. and termed socketing.^67^ Socketing might be advantageous to delay common nanoparticle deactivation mechanisms such as particle detachment and agglomeration during operation.^33^ The particles of STFNO Ex.600 °C appear to be homogeneous, while the particles of STFNO Ex.800 °C exhibit a core surrounded by a 2 nm thick surface layer—shell—(Figure 4F). This finding is similar to what Zhu et al. previously reported. However, they observed an amorphous surface layer, whereas we observed a crystalline surface layer (Figures 4F and S8).^62^

EELS was used to determine the composition of the exsolved particles. The EELS spectra of the STFNO - Ex.600 °C in Figure 4I, top displayed a Ni to Fe ratio of 1.3, whereas other analyzed particles shown in Figure S6 and Table S2 revealed higher ratios of 2.2 and 2.7. It should be noted that the oxygen edge was not included in the EELS analysis, but the particles are expected to be oxidized. The following subsection presents the oxidation behavior of exsolved particles as observed during ETEM experiments. HRTEM analysis of STFNO - Ex.600 °C (Figure S7) showed lattice spacings of 2.1 Å, consistent with NiO fcc (200). It should be noted that fast buildup of carbon during the measurement made it difficult to record STEM pictures of the analyzed particles (see Figure 4G).

The core–shell structured nanoparticles of STFNO - Ex.800 °C unveiled a significant difference between core and shell composition: EELS analysis of a particle (shown in Figure S9) shows that the core exhibited a metal ratio of 44 atom % Fe and 56 atom % Ni, similar to that of the nanoparticles of STFNO - Ex.600 °C, whereas the shell displayed a composition of 84 atom % O and 16 atom % Fe. To confirm this result, a linescan through another exsolved particle of STFNO - Ex.800 °C was carried out, and EELS spectra were measured at 10 different positions with increasing depth through the particle (indicated by the color gradient in Figure 4H/I). Core-loss spectra in the region of transition metals and the O–K edge exhibited a clear trend: the O–K peak decreased when scanning from the shell into the core, while the Ni–L_3,2_ peak appeared when scanning into the core. The Fe–L_3,2_ peak is present in both the core and shell of the particle. All signals of O–K, Fe–L_3,2_, and Ni–L_3,2_ are observed upon scanning into the host structure. However, the peak intensity of all three is lower when scanned into the host structure due to the increasing sample thickness. Conforming core-shell structuring of the nanoparticles exsovled at 800 °C. The lattice parameters of STFNO Ex.800 °C determined from FFT HRTEM images (see Figure S8) exhibited a core *d*-spacing of 2.1 Å, matching that of NiO fcc (200), and a shell *d*-spacing of 2.55 Å, matching that of Fe_2_O_3_ fcc (311). However, other materials also match this *d*-spacing (Table S3).

### Oxidation of Exsolved Particles

To understand the evolution of the exsolved particles, in situ exsolution was performed. Below 600 °C, no exsolved particles were observed on the surface of STFNO, even after 1 h of waiting time at 600 °C. Upon increasing the temperature to 650 °C, nanoparticles were visible all over the host, as shown in Figure 5A. After 5 h, the particles reached a size of 3.4 ± 0.5 nm; see Figure 5B. Afterward, the sample was cooled to room temperature, removed from the ETEM, and exposed to air. After a 0.5 h waiting time, the sample was reinserted into the ETEM, and the same positions were analyzed; see Figure 5C. The particles have visually grown in size to 7.6 nm, matching the size of the particles ex-situ exsolved at 600 °C. The growth seems to match the oxidation of the particles, as shown in Figure S11. Here, the size is calculated assuming different crystal structures after air exposure using the amount of exsolved Ni and Fe. This confirms that the ex-situ exsolved particles are first metallic particles inside the ETEM during exsolution and are then oxidized and grown in size when exposed to air (see schematic in Figure 5D).

**Figure 5:**
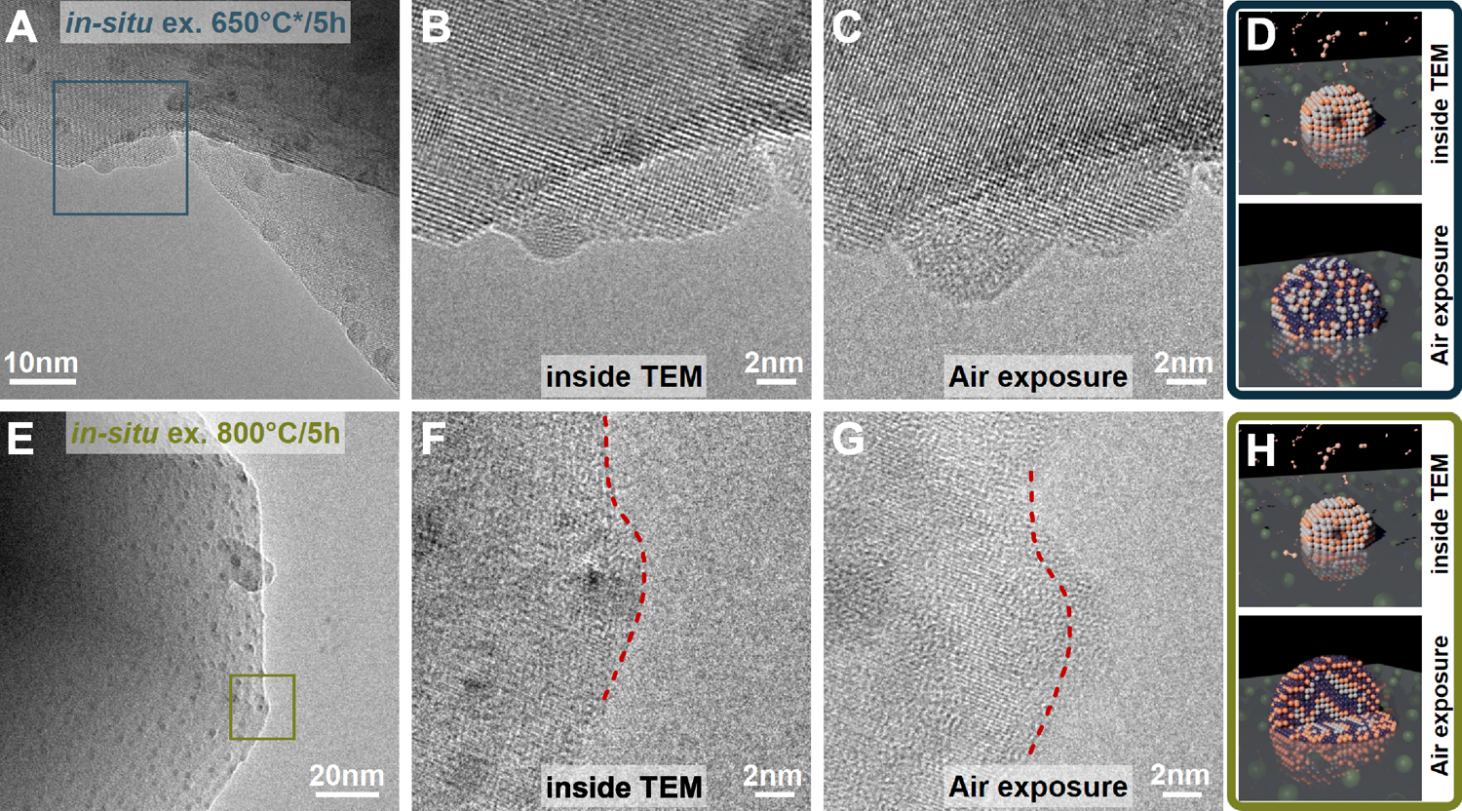
In situ exsolution experiment inside ETEM at (A–C) 650 °C and (E–G) 800 °C (A) overview picture after 5 h (B) detailed region after 5 h inside ETEM (C) detailed region after air exposure and reinsertion (D) schematic of particle oxidation (top) first mixed FeNi-particle then (bottom) oxidized particle (E) overview picture after 5 h (F) detailed region after 5 h inside ETEM (the red dashed line indicated the host structure) (G) detailed region after air exposure and reinsertion (the red dashed line indicated the host structure) (H) schematic of particle oxidation (top) first mixed FeNi-particle then (bottom) oxidized core–shelled particle.

### Core–Shell Formation

A similar in situ experiment was performed; however, this time the sample was directly heated to 800 °C with the same heating rate as during the ex-situ experiment. The results can be seen in Figure 5E/F. The formed particles are slightly larger in size 3.9 ± 0.3 nm and are homogeneous at the end of the 5 h hold at 800 °C. Afterward, the sample holder is cooled to room temperature, removed from the ETEM, and exposed to air overnight. Upon reinsertion, it is possible to observe a faint core–shell structure, as shown in Figure 5G. This indicated that the core–shell structure formed during air exposure and is probably related to the composition of the exsolved particle, as ex-situ experiments have shown more Fe-rich particles when exsolution is performed at higher temperatures. The pseudobinary phase diagram of Fe_2_O_3_–NiO in air predicts that Fe-rich compositions will separate into Fe_2_O_3_ and (FeNi)_3_O_4_, whereas Ni-rich compositions will form homogeneous oxides (FeNi)_3_O_4_ and (FeNi)­O.^68^ This could be the reason for the formation of the core–shelled particle, as shown by schematic [Fig fig5]H.

### Activity and Stability of STFNO with Exsolved Nanoparticles

The electrochemical results after exsolution are shown in Figure 6. The cathodic sweeps of STFNO - Ex.600 °C tested at 50 bar in 10 M KOH can be seen in Figure 6A and display continuous thermal activation as the temperature is raised from 25 to 75 °C. The cathodic sweeps exhibit a reduction feature at approximately 1.25 V vs RHE. The position indicates that the feature is associated with the Ni^3+^/Ni^2+^ redox transition. As this feature is not visible in the STFNO sample, it is most likely caused by the presence of the exsolved Ni-rich nanoparticles.

The overpotential required to draw 10 mA cm^–2^ at 50 bar in 10 M KOH at different temperatures for the STFNO, STFNO - Ex.600 °C, and STFNO - Ex.800 °C samples is compared in Figure 6B. Exsolution resulted in a decrease in overpotential throughout the displayed temperature range. For instance, STFNO Ex.600 °C exhibits an overpotential at 10 mA cm^–2^ at 25 °C of 306 ± 41 mV, 231 ± 12 mV at 50 °C, and 199 ± 15 mV at 75 °C. In comparison, the overpotential of STFNO Ex.800 °C is slightly lower at 25 °C with 284 mV; however, at 50 and 75 °C, the overpotential is somewhat higher at 251 and 224 mV, respectively. The differences are most likely caused by the difference in nanoparticle density and composition, as STFNO - Ex.600 °C displayed mixed Ni_*x*_Fe_*y*_O_*z*_ particles and STFNO - Ex.800 °C displayed core–shell structured nanoparticles with a Fe_2_O_3_ shell.

To examine the stability of the nanoparticles, STFNO - Ex.600 °C was tested up to 75 °C or up to 100 °C at 50 bar in 10 M KOH for approximately 10 h. The post-mortem analysis showed that the nanoparticles after testing at 75 °C had doubled in size (see Figure 6D) compared to the pristine nanoparticles. When tested at 100 °C, the particles had disappeared from the surface; see Figure 6E. A schematic illustration of the nanoparticle stability is shown in [Fig fig6]F: (1) the sample is uniformly decorated with exsolved nanoparticles. (2) After testing for 10 h at 75 °C, the nanoparticles have grown in size, and their density has decreased. The observed growth in nanoparticle size may reflect electrolyte-driven processes of partial dissolution and redeposition of Fe and/or Ni. (3) After testing at 100 °C, the particles have disappeared. The overpotential decrease from 75 to 100 °C in Figure 6B is indicated with a dashed line to mark the stability limit of the exsolved particles. Testing of STFNO - Ex.800 °C up to 100 °C also showed that no nanoparticles were present after the test (see Figure S12). This indicates that the stability of the exsolved nanoparticles is limited to temperatures below 100 °C. It is worth noting, though, that even after the loss of the exsolved nanoparticles, the catalyst retains substantially improved performance compared to the pristine STFNO. The absence of visible nanoparticles after testing at 100 °C could also be due to the particles being below the detection limit of the SEM. Furthermore, the remaining improved performance could be due to the formation of an active Ni,Fe oxy­(hydroxide) layer on the surface of the perovskite. However, we do not have direct evidence of the formation of such a layer from XPS, XRD, or SEM. It should also be noted here that the measured cell resistance is significantly lower for the exsolved sample compared to the pristine material, and notably, this reduced resistance remains stable even after the nanoparticles are no longer visible. This indicates a lasting modification of the perovskite following exsolution and OER testing. The exsolution conditions are expected to alter the perovskite defect chemistry, resulting in increased electronic conductivity.^69–71^ The bulk defect chemistry is expected to remain frozen below approximately 500 °C, i.e., at temperatures where the OER is taking place, in line with the retained improved conductivity.^71,72^ This modification of the electronic structure of the perovskite may also be responsible for the improved OER activity observed after exsolution, which would explain why the activity remains unchanged even after the disappearance of the exsolved nanoparticles following OER testing at 100 °C.

**Figure 6:**
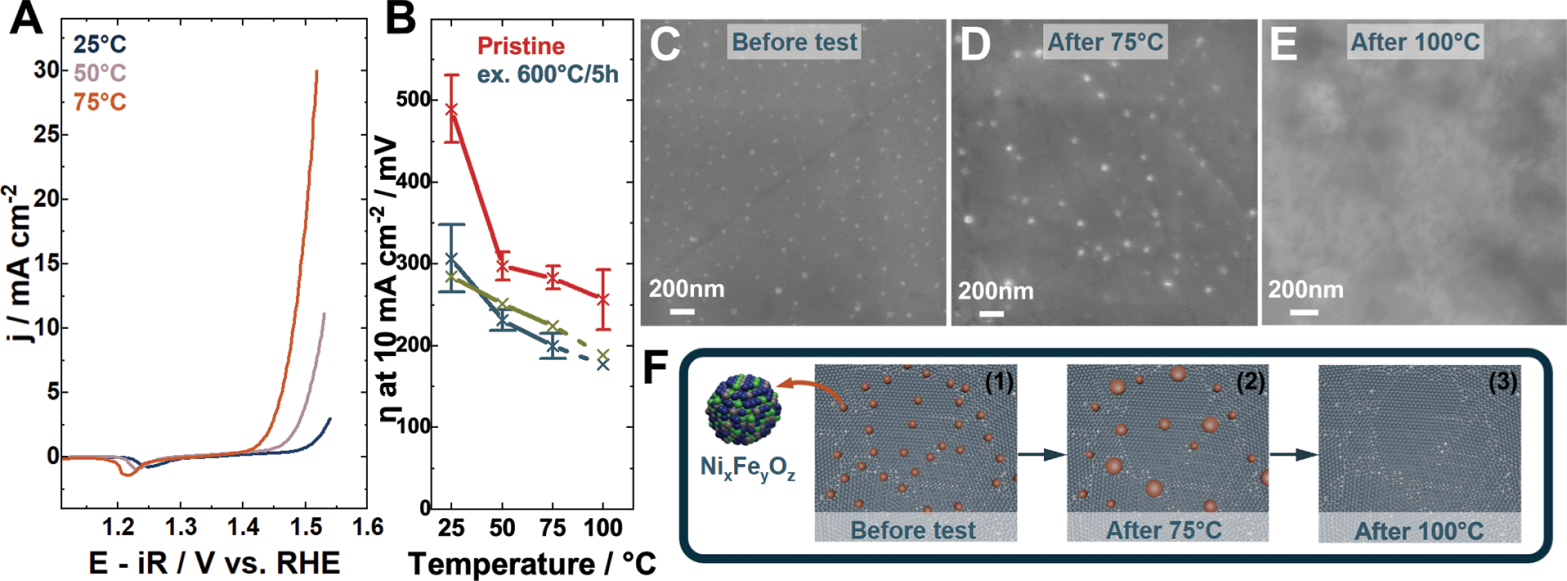
OER activity and stability of STFNO with exsolved nanoparticles at 25–100 °C, 50 bar, 10 M KOH. (A) Cathodic sweeps at a scan rate of 10 mV s^–1^ for STFNO - Ex.600 °C. (B) Overpotential development at the benchmark current density of 10 mA cm^–2^ (with average of two measurements and the error bar representing the standard deviation of the mean for STFNO - Ex.600 °C. (C) SEM micrograph after exsolution at 600 °C. (D) SEM micrograph after testing at 75 °C. (E) SEM micrograph after testing at 100 °C. (F) Schematic illustration of nanoparticle stability (1) initial (2) coarsened particles (3) no particles left.

## Conclusions

The scarce testing of OER electrocatalysts for AEL under industrial conditions represents a critical gap between academic research and industrial development, with recent studies suggesting the poor stability of, e.g., the intensively researched (Ni,Fe)­OOH OER catalyst at industrial conditions. Perovskite-based OER catalysts have gained attention as alternatives, yet the most commonly investigated compositions also show instability under industrial conditions. In this study, we present an alternative perovskite, STFNO, with good activity and stability under industrial conditions and beyond. STFNO exhibits high activity at 50 bar in 10 M KOH, with an overpotential for 10 mA cm^–2^ of 283 mV at 75 °C, which further decreases to 183 mV at 150 °C. The material undergoes a temperature-induced surface activation at the start of the test at 50 °C, likely associated with exposure of highly active surface sites. STFNO showed excellent stability at 100 °C for 200 h, stabilizing at an overpotential of 320 mV and revealing a nearly intact microstructure, albeit with an increased A to B ratio and increased Ni amount on the B-site. However, at 150 °C, STFNO exhibits a more pronounced modification, with surface roughening and particle detachment. Furthermore, we observed a substantial pressure dependency of the OER activity at room temperature, highlighting the importance of testing under industrially relevant conditions.

Exsolution at different temperatures produced evenly distributed nanoparticles at the surface of the host, with homogeneous particles, when exsolved at 600 °C, and core–shelled particles, when exsolved at 800 °C, enhancing the OER activity by 30% compared to STFNO. Notably, STFNO with nanoparticles exsolved at 600 °C resulted in the lowest overpotential for 10 mA cm^–2^, such as 306 mV at 25 °C and 199 mV at 75 °C. Despite these benefits, the nanoparticles showed instability during short-term testing at 100 °C, indicating a limitation in their practical application in AEL but of interest for anion exchange membrane electrolysis at lower temperatures.

In conclusion, doped STO, with or without exsolution, emerges as a very promising CRM-lean OER catalyst option in an alkaline environment due to its high activity and stability at temperatures up to at least 100 °C. Furthermore, it offers a very diverse platform, with many alternatively doped SrTiO_3_ and their exsolved counterparts representing promising systems for further exploration as OER catalysts. In addition, several questions remain unresolved, relating to the reaction mechanisms, the rate-determining steps, possible synergy between host and exsolved phases, and the evolution of surface chemistry during operation, which are all critical for advancing our understanding of these systems. Addressing these will facilitate the rational optimization of these catalysts for industrial applications.

## Experimental Section

### Material Synthesis and Preparation

Sr_0.95_Ti_0.95–*x*_Fe_*x*_Ni_0.05_O_3_, with *x* = 0.05; 0.25; 0.45; 0.65, and Sr_0.98_Ti_0.7_Fe_0.25_Ni_0.05_O_3_ have been synthesized using a conventional solid-state reaction. Stoichiometric ratios of SrCO_3_, TiO_2_, Fe_2_O_3_, and Ni­(NO_3_)_2_6H_2_O were dispersed in ethanol and ball-milled for 24h. Afterward, the powder was dried, pressed into a large pellet of 30 mm, and calcined at 1050 °C for 10 h. Thereafter, the material was crushed by high-energy ball milling for 5 min and uniaxially pressed with 215 MPa two times for 45 s into pellets with a diameter of 12 mm and a thickness of approximately 2 mm. The pellets were sintered in air at 1300 °C for 10 h, with a heating and cooling rate of 1.5 C min^–1^. Subsequently, the samples were polished using a semiautomatic polishing machine, Struers Tegramin, using a diamond suspension of 0.25 μm. Exsolution was performed in a 4% H2/96% N2 atm at different temperatures from 600 to 800 °C for 5 h, with a heating and cooling rate of 1.5 C min^–1^.

### Physical Characterization

Powder XRD analysis was conducted using a Panalytical Aeris diffractometer equipped with a Cu–Kα X-ray source. Data collection spanned from 20 to 80° in 2θ, with a step size of 0.01° in 2θ. FEGSEM analysis was performed with a Zeiss Merlin instrument operating at 5 kV and a probe current of 129 pA. Nanostructure and composition data were obtained using a probe-corrected Titan Analytical in STEM at 300 kV, equipped with an Oxford windowless EDS detector and a GIF for electron energy loss spectroscopy (EELS). In situ ETEM experiments were performed using an ETEM instrument (FEI, Titan 80–300 kV) equipped with a differential pumping system, where the sample was exposed to H_2_ with a pressure of 4.1 mbar. The sample ink was dropcasted onto a MEMS chip (DENS Wildfire holder). The heating profiles can be found in Figure S10. Particle size and distribution were analyzed by utilizing ImageJ software. The lattice parameter was determined using CrystalMatch software by Simomsen et al.^73^ XPS analysis was carried out using a Thermo Scientific Escalab 250Xi XPS instrument with an Al–Kα source (1486.6 eV), operated in normal emission mode at 15 kV and 10 mA. The spectrometer binding energy scale was calibrated by using the Au 4f peak at 84 eV prior to sample analysis. Sr 3d, Ti 2p, Fe 2p, Ni 2p, and O 1s narrow scan spectra were recorded at a pass energy of 30 eV, and survey spectra were recorded at a pass energy of 100 eV. Inbuilt charge compensation was utilized during measurement, and the resulting spectra were charge-corrected to the adventitious carbon signal at 285.0 eV eV. Core-level spectra were deconvoluted using CasaXPS software with Shirley-type background correction and 30% Gaussian, 70% Lorentzian line-shapes (GL30). ICP-OES analysis was performed with an Agilent 5800 instrument.

### Accelerated Immersion Stability Test

An accelerated immersion stability test was conducted to evaluate the chemical stability of the catalyst materials similar to Buchauer et al.^17^ For this procedure, 0.5 g of the perovskite powder was placed in 10 M concentrated KOH inside a 50 mL autoclave with a PTFE insert, which was filled to about two-thirds of its volume. The autoclave was heated in a furnace at 200 °C for 72 h. Afterward, the material was cooled to room temperature, vacuum filtered, and rinsed with 1 *l* of hot water. The sample was then centrifuged in water and dried in air at 80 °C. Finally, the powder was subjected to XRD analysis.

### Electrochemical Characterization

The electrochemical performance was evaluated using the test setup described in previous articles.^4,7,74^ For testing the dense pellets, the same setup as in Buchauer et al. was used.^17^ For all experiments, a perforated Ni plate served as the counter electrode. This electrode was pretreated by immersion in 1 M HCl for 15 min, followed by washing and sonication in water for 5 min. A porous YSZ ceramic was employed as the separator.^7^ The sample was mounted in the holder and contacted with the Pt current collector. Expanded PTFE was used as a gasket material and placed in front of the pellet to ensure a well-defined test area of 0.28 cm^2^. For the test, a beaker cell with a volume of 25 mL filled with 10 M concentrated KOH was used. As a reference electrode, a homemade Pt-RHE, similar to Leuaa and Chatzichristodoulou, was placed behind the counter electrode and calibrated against the lab-standard prior to each test.^75^

Testing was performed in 25 K intervals, starting from 25 to 150 °C; however, some experiments were intentionally stopped at a lower test temperature. All tests were carried out at a pressure of 50 bar. Prior to pressurization, the electrochemical active surface area (ECSA) was measured using potential cycling at various scan rates in the potential region from 1.05 to 1.15 V vs RHE, and a full characterization was carried out.

A full characterization consists of the following protocol: first, the ohmic resistance was measured using potentiostatic impedance at 1.2 V vs RHE. The material was then stabilized by performing cyclic voltammetry within the potential range of 1–1.7 V for 100 cycles. Following this, linear sweep voltammetry was conducted at a scan rate of 10 mV s^–1^, first in the anodic and then in the cathodic direction in the same potential range. Chronopotentiometry measurements were then carried out for 5 min at current densities of 50, 20, 10, 5, 2, and 1 mA cm^–2^. The chronopotentiometry at 10 mA cm^–2^ was followed by galvanostatic impedance at the same current density, with a 5% amplitude, covering 12 *points* per decade from 200 kHz to 0.1 Hz. Finally, another potentiostatic impedance measurement was performed at 1.2 V vs RHE.

The autoclave was heated at a rate of 2 K min^–1^, and the cell was allowed 90 min to stabilize at the respective temperature. Then, a full characterization was carried out. After cooling to room temperature with sufficient waiting time, the ECSA was determined again, and a full characterization was carried out.

Stability testing was performed by testing in 25 °K intervals to the stability testing temperature of 100 or 150 °C. Then, a full characterization was performed. Afterward, the material was galvanostatically held at 10 mA cm^–2^ for 200 h. Subsequently, a full material characterization was carried out at the stability test temperature.

All of the presented data are 100% *iR*-compensated to offset for the solution resistance, wires, and electronic transport through the dense polished pellets. The error bars presented here are from multiple measurements of different samples of the same composition. The overpotentials presented in this work are calculated using the formulas from Balej to account for the temperature and pressure dependence of the oxygen and hydrogen equilibrium potential in an aqueous KOH solution.^76,77^

## Supplementary Material



## Data Availability

Data for this article, including all electrochemical data and images, are available at DTU data at 10.11583/DTU.28282172.

## Notes

XRD data of the pristine sample, additional electrochemical data such as CV at different temperatures and overpotential at 1 and 50 bar, additional SEM pictures of untested and tested samples, and additional TEM images of the different exsolved samples (PDF)
