# Supplementary material for: Stability and Exsolution of Sr0.98Ti0.7Fe0.25Ni0.05O3 for the Oxygen Evolution Reaction in an Alkaline Environment
Source: J Am Chem Soc. 2025 Jul 16;147(30):26402–13. doi: 10.1021/jacs.5c05748 (PMC12314905; doi:10.1021/jacs.5c05748)
Supplement: Supplementary file 1 [file ja5c05748_si_001.pdf]

Supporting Information

Stability and Exsolution of $Sr_{0.98}Ti_{0.7}Fe_{0.25}Ni_{0.05}O_3$ for the Oxygen Evolution Reaction in Alkaline Environment

Fabian Luca Buchauer,[*,†] SangWoo Kim,[‡] Søren Bredmose Simonsen,[†] Roxy Lee,[†] WooChul Jung,[‡,¶] and Christodoulos Chatzichristodoulou[†]

†*Department of Energy Conversion and Storage, Technical University of Denmark (DTU), Building 310, Fysikvej, Lyngby 2800, Denmark*

‡*Korea Advanced Institute of Science & Technology (KAIST), 291 Daehak-ro, Yuseong-gu, Daejeon 34141, Republic of Korea*

¶*Seoul National University (SNU), 1, Gwanak-ro, Gwank-gu, Seoul 08826, Republic of Korea*

E-mail: falubu@dtu.dk

XRD results of samples before accelerated stability testing

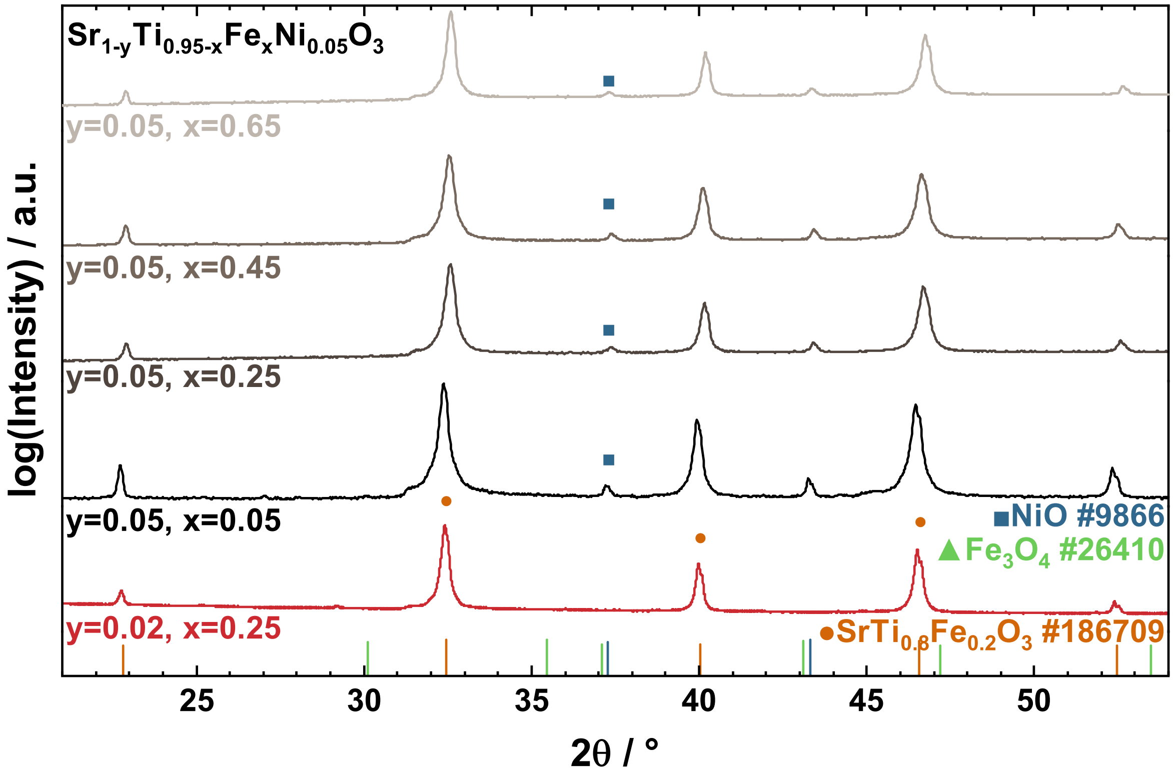

Figure S1: XRD of pristine samples.

Activation CVs

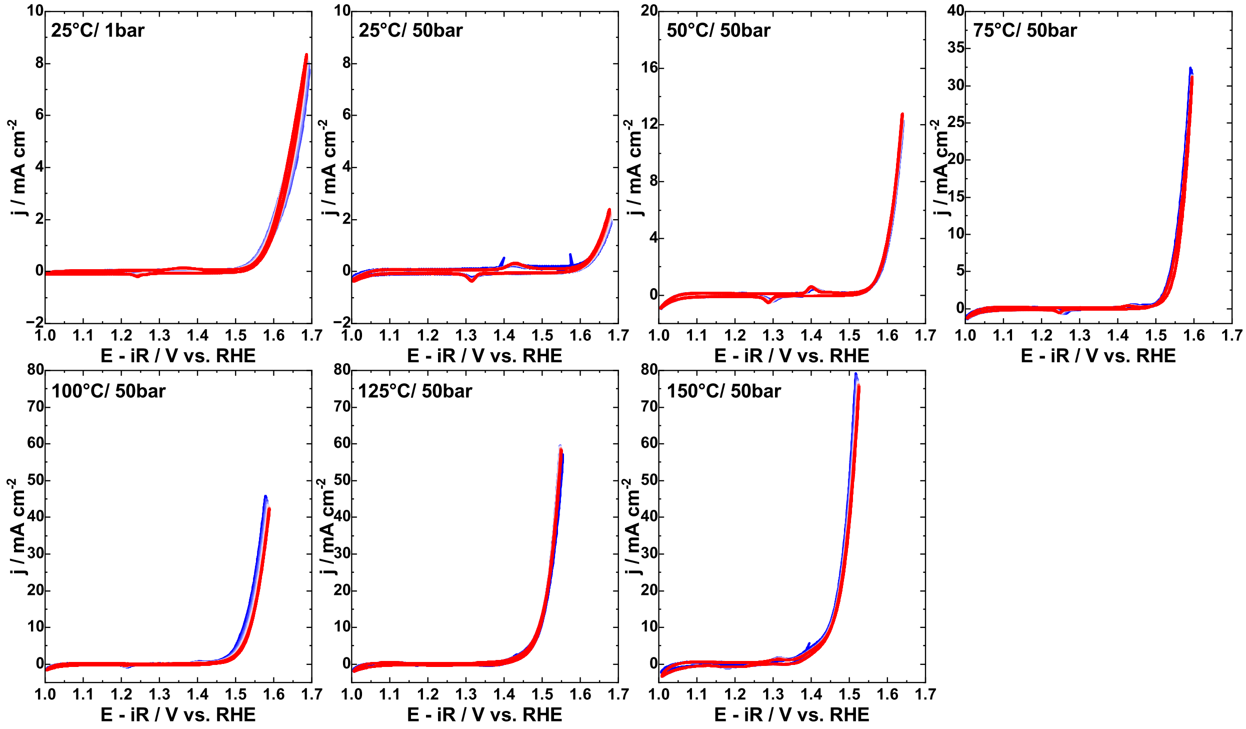

Figure S2: Activation with CV cycling at $100\,\mathrm{mV\,s^{-1}}$ of STFN (gradient from blue to red with blue being a lower cycle number).

Overpotential comparison at 25 °C

Table 1: Overpotential at 25 °C at 1 bar and 50 bar.

Sample	1 bar / mV	50 bar / mV
1	517	474
2	484	464
3	660	592
4	569	513
5	413	402

SEM of pristine sample

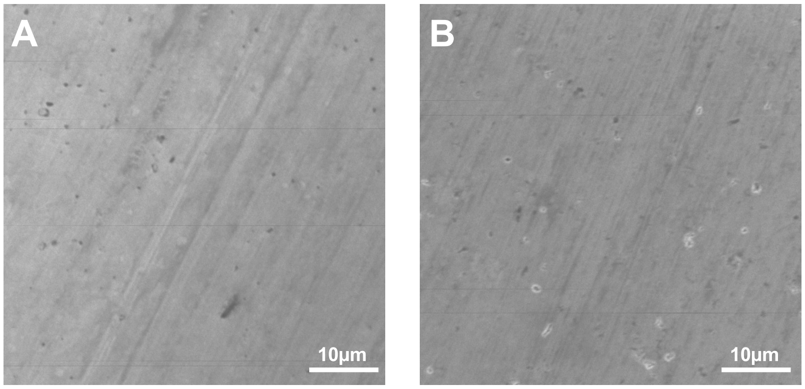

Figure S3: SEM of pristine STFNO A) before 100 °C B) before 150 °C.

SEM pictures of post mortem 100 °C

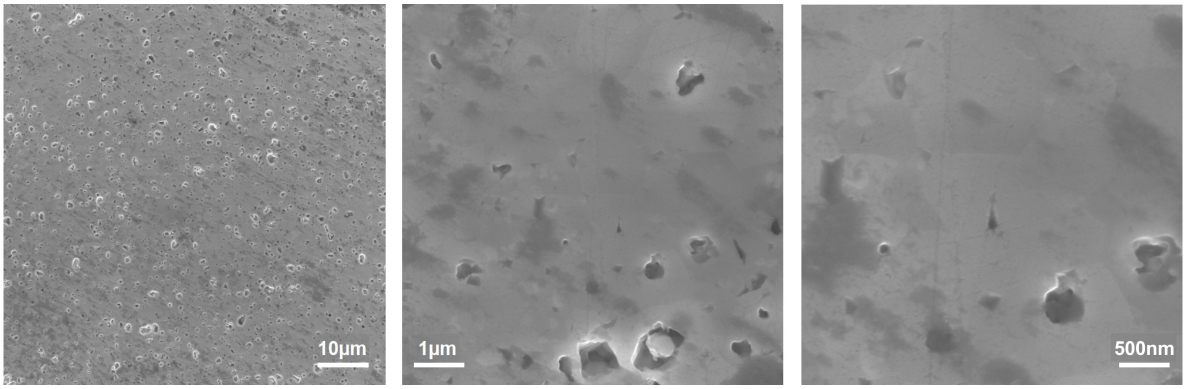

Figure S4: SEM of post mortem STFNO 100 °C.

SEM pictures of post mortem 150 °C

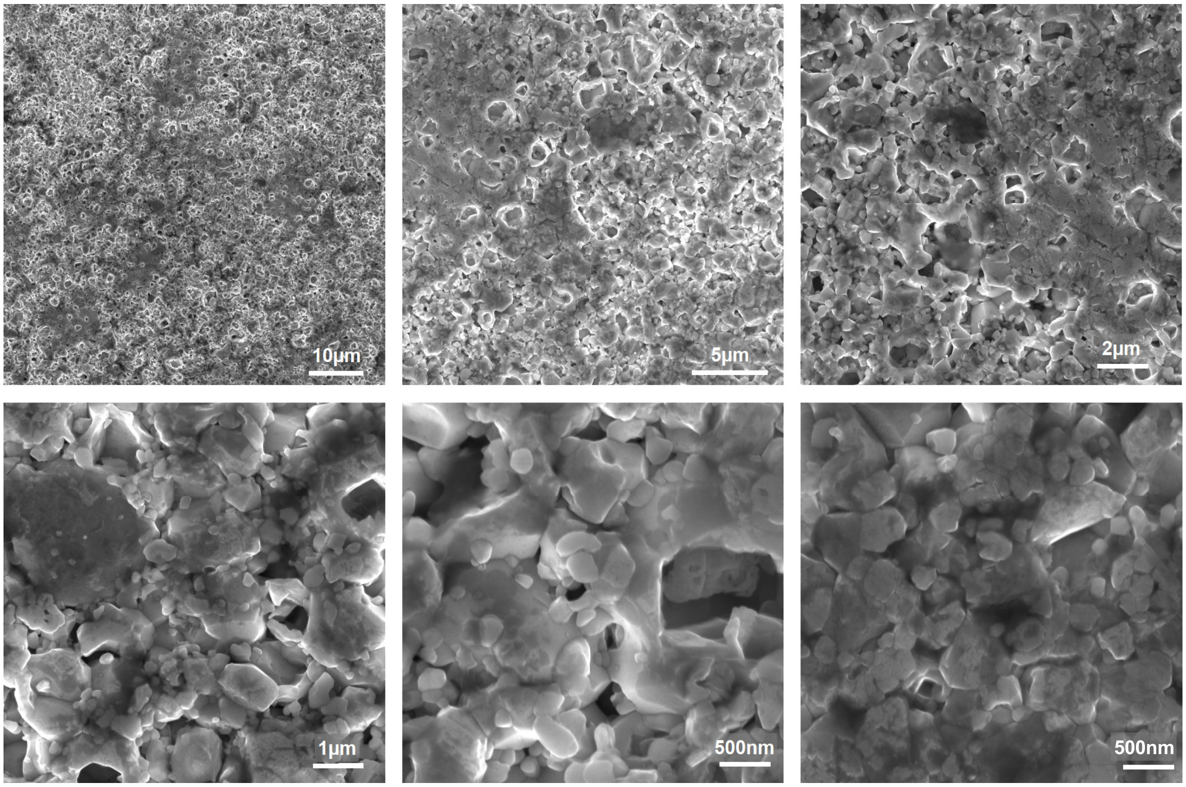

Figure S5: SEM of post mortem STFNO 150 °C.

Particles with different Fe to Ni ratio exsolved at 600 °C

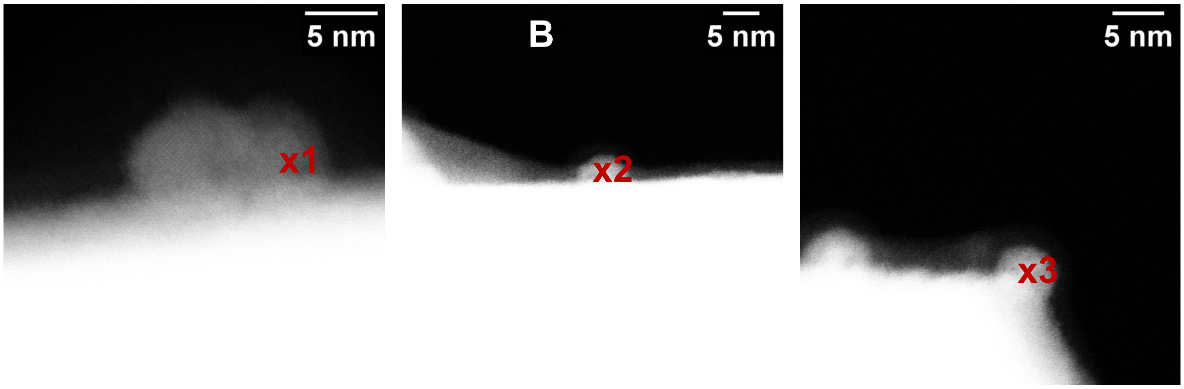

Figure S6: Particles with different Fe to Ni ratio exsolved at 600 °C.

Table 2: EELS composition of particles ex-situ exsolved at 600 °C.

	Ni / at.%	Fe / at.%
1	57	43
2	73	27
3	69	31

Exsolution at 600 °C

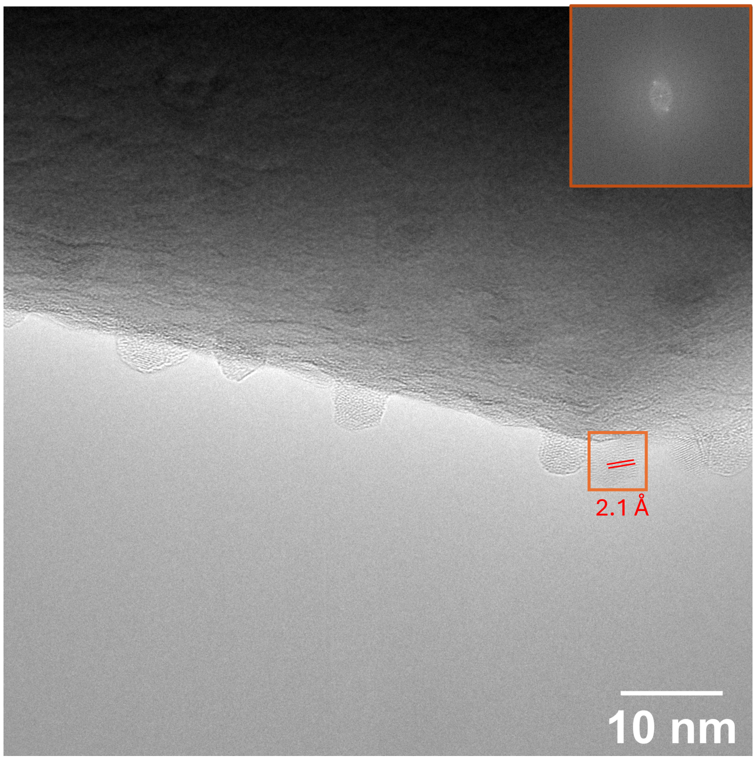

Figure S7: Homogeneous particles exsolved at 600 °C.

Exsolution at 800 °C

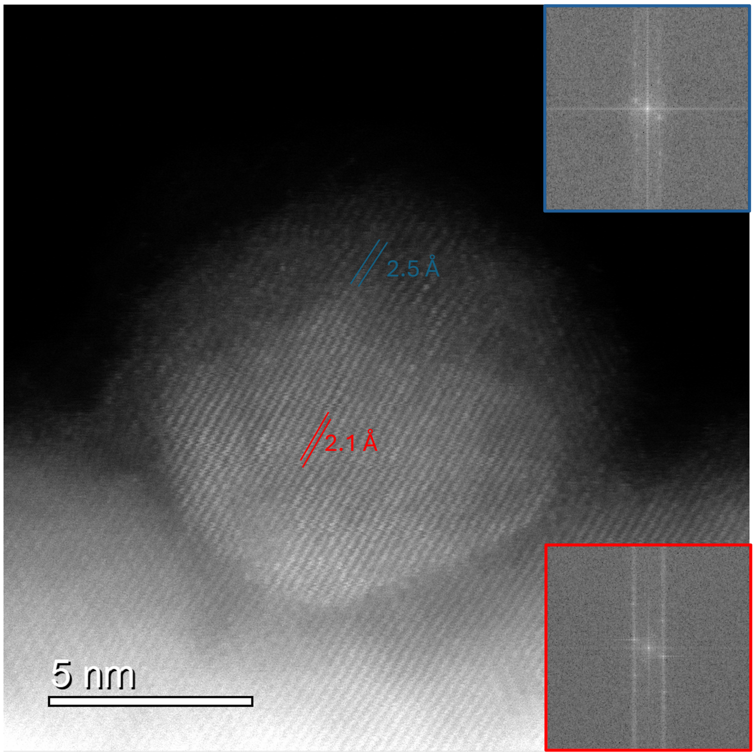

Figure S8: Core-shelled particle exsolved at 800 °C showing a crystalline shell.

Table 3: Lattice parameters of relevant Fe and Ni compounds.

Compound	Lattice parameter / Å
Fe_2O_3 FCC (311)	2.53
Fe_2O_3 trigonal (2-10)	2.52
NiO FCC (111)	2.41
NiO_2 trigonal (006)	2.39
NiO FCC (200)	2.09
Fe_2O_3 FCC (400)	2.10
Fe_2O_3 trigonal (311)	2.21

EELS particles used for core-shell quantification

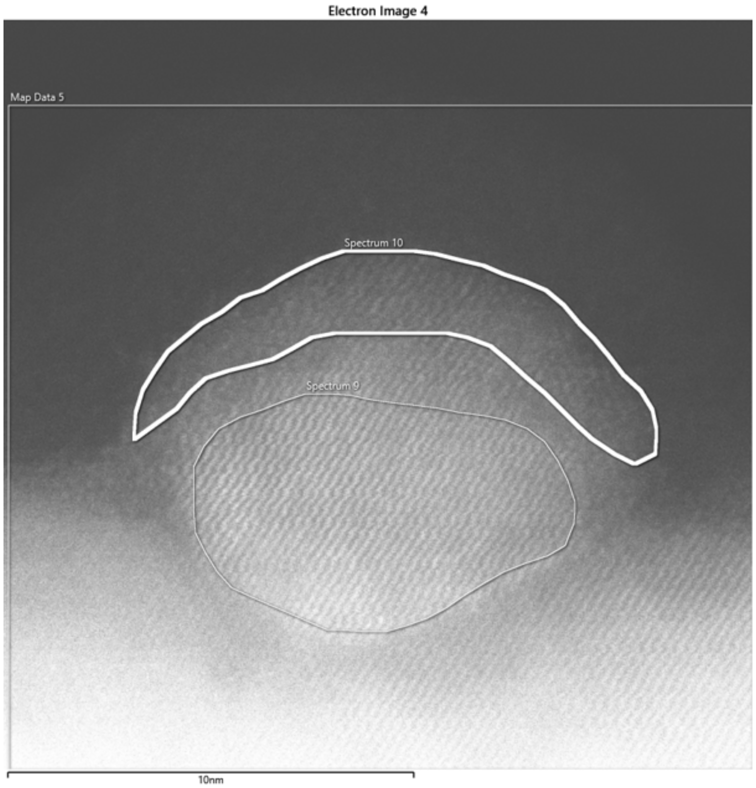

Figure S9: Areas for core-shell quantification.

Heating curves for *in-situ* experiments

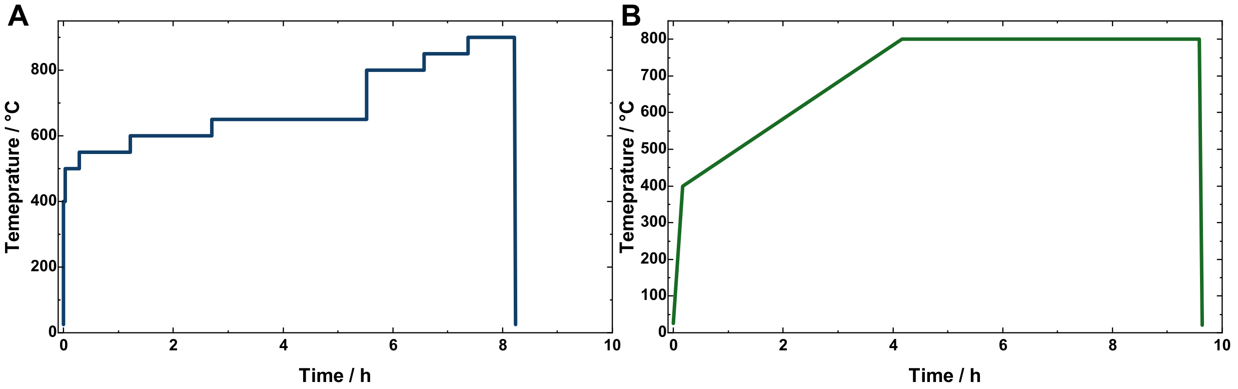

Figure S10: Temperature profile for the two *in-situ* experiments A) oxidation experiment B) core-shell experiment.

Oxidation of particles

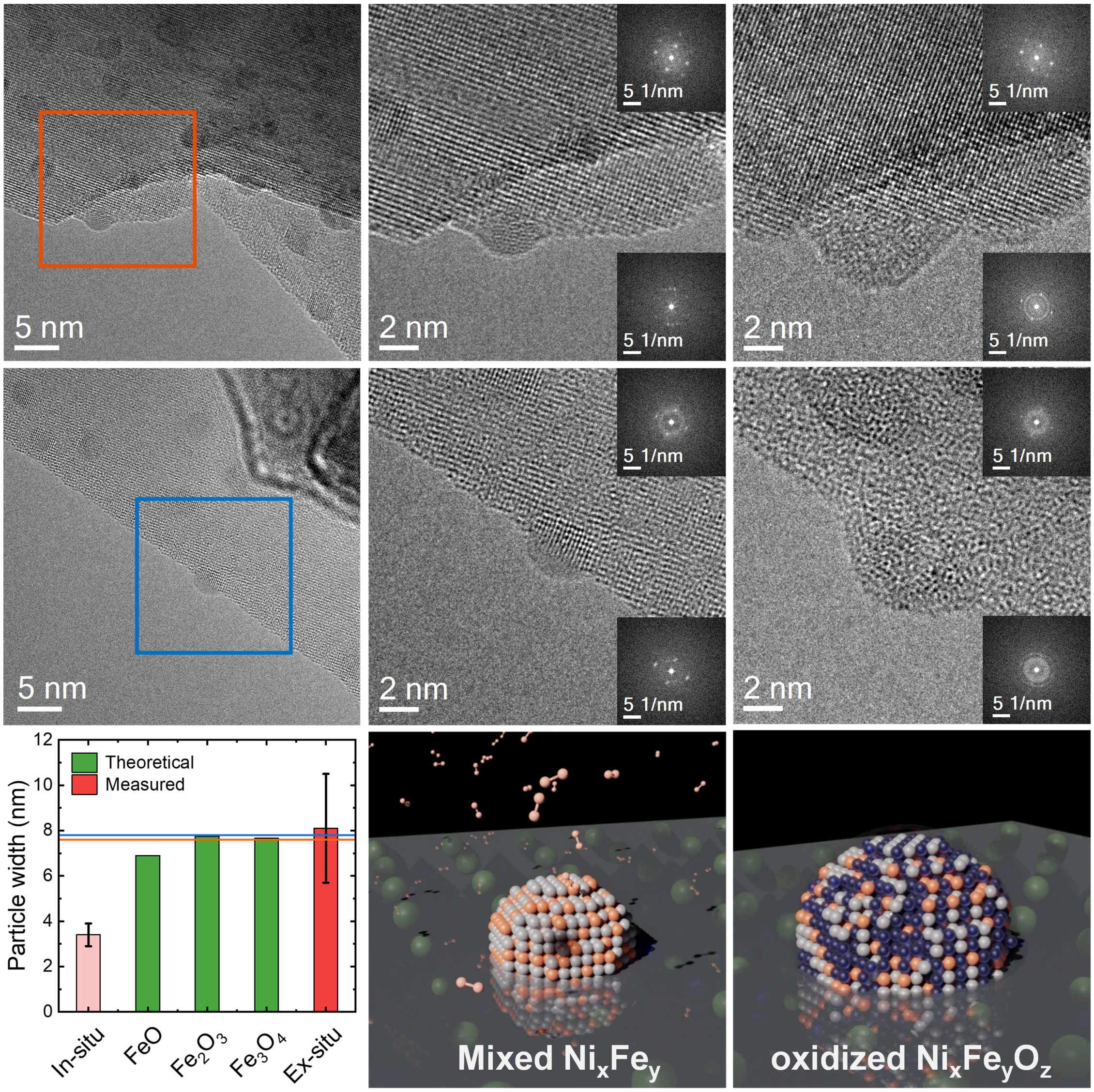

Figure S11: Particle oxidation for 2 different regions and calculation of growth assuming oxidized crystal structure.

Exsolution 800 °C sample after test

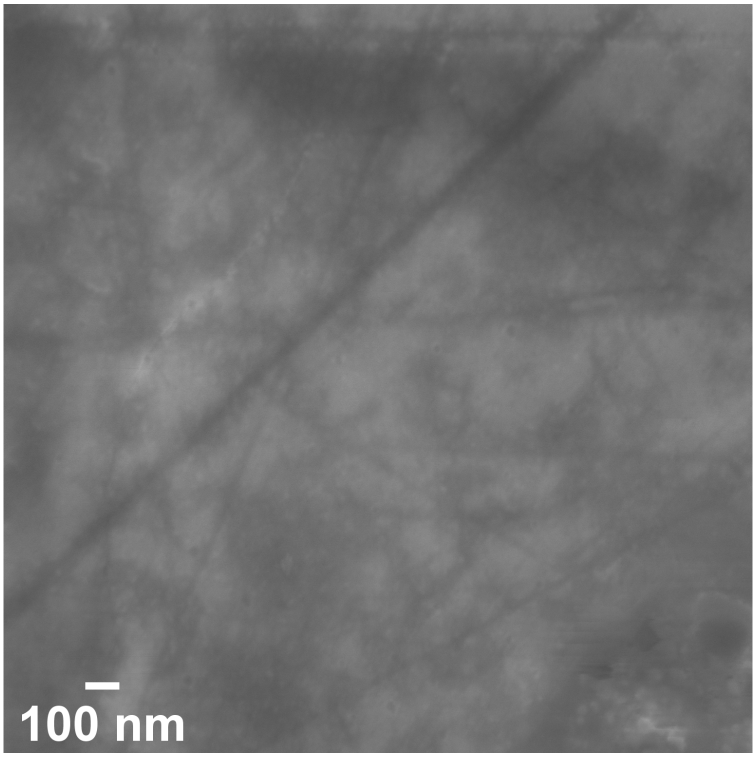

Figure S12: Exsolution 800 °C sample after test.

XPS raw data

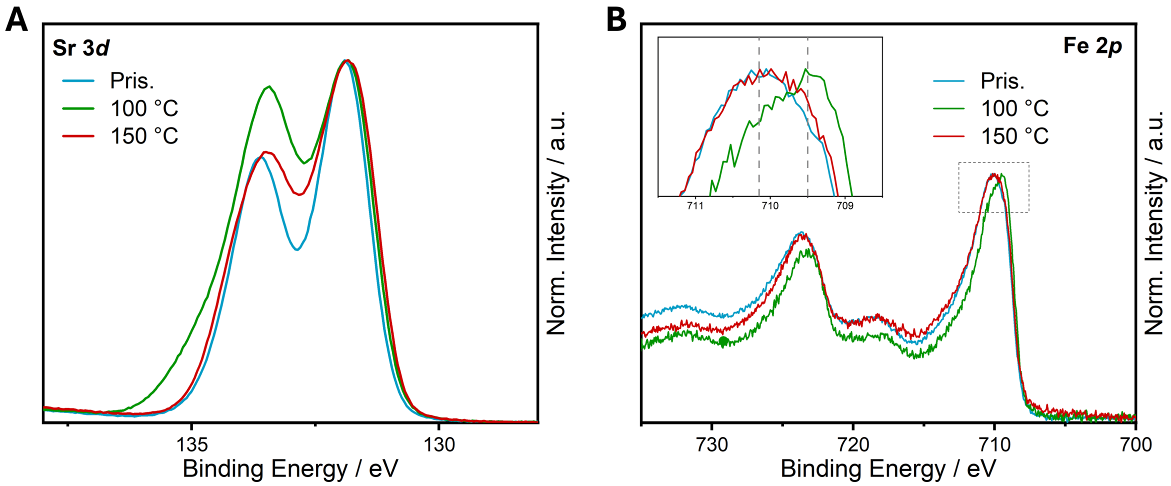

Figure S13: A) Overlay of Sr 3d data B) Overlay of Fe 2p data.
